# Supplementary material for: Pathological Features in Paediatric Patients with TK2 Deficiency
Source: Int J Mol Sci. 2022 Sep 20;23(19):11002. doi: 10.3390/ijms231911002 (PMC9570075; doi:10.3390/ijms231911002)
Supplement: Supplementary file 1 [file ijms-23-11002-s001.zip › ijms-1887978-supplementary.pdf]

Case	1	2	3	4	5	6	7	8
Sex	Female	Male	Female	Male	Female	Male	Male	Male
Age at debut (months)	9 m	12 m	14 m	17 m	23 m	30 m	32 m	36 m
Age at biopsy (months)	10 m	16 m	17 m	21 m	27 m	108 m	48 m	180 m
Clinical phenotype	Myopathic	Myopathic	Myopathic	Myopathic	Myopathic	Myopathic	Myopathic	Myopathic
First symptom	Axial hypotonia and progressive weakness, specially “floppy head”	Hypotonia, proximal weakness	Hypotonia, proximal weakness,	Hypotonia. Progressive weakness	Hypotonia and delayed motor milestones	Facial, proximal and axial muscle weakness	Fall to the ground. Dificulty walking	Dystal weakness
Cognitive delay	No	No	No	No	No	No	No	No
Ambulatory	No	No	No	No	Yes	No	Yes	Yes
Age when lost ability to walk		20 m	36 m	22m		10 years		
Ptosis/Opthalmoplegia	No	No	No	No	No	Yes/No	No	Yes/No
Facial Weakness	Yes	No	No	No	No	Yes	No	Yes
Weakness	Axial/proximal/distal	Axial/proximal	Axial/proximal	Axial/proximal	Axial/proximal	Axial/proximal/distal	Axial/proximal	Axial/proximal/distal
Mechanical ventilation	Yes	Yes	Yes	Yes	No	Yes	Yes	Yes
Failure to thrive	n.a.	Yes	Yes	No	No	Yes	No	Yes
Dysphagia	Yes	Yes	Yes	Yes	No	Yes	Yes	Yes
Feeding tube	Yes (NGT)	Yes (NGT)	Yes (PEG)	Yes (NGT/PEG)	No	Yes (NGT)	Yes (NGT)	No

Date started therapy	2015	2018	2019	2014	2015	2016	No	2015
Death	No	Yes	No	No	No	No	Yes	no
CPK (Reference values)	2.071 UI/l (<228)	663 UI/L (< 174)	881 IU/L (<174)	274 UI/L (< 190)	1.434 UI/L (<220)	538 UI/L (< 190)	540 UI/L (< 190)	160 UI/L (< 190). No at onset)
GDF15	n.a.	14.156 pg/mL	3.510 pg/mL	14.756 pg/mL	15.000 pg/mL	4.608 pg/mL	n.a.	n.a.
Mutation in *TK2* gene	c.[323C>T];[676_677dup]	c.[323C>T];[360_361delinsAA]	c.[360_361delinsAA];[416C>T]	c.[388C>T];[623A>G]	c.[604_606delAAG];[529G>T]	c.[360_361delinsAA]; [360_361delinsAA]	c.[c.604_606delAAG];[150dupA]	c.[388C>T]; [604_606delAAG]
Mutation in TK2 protein	p.[Thr108Met];[Pro227Serfs*9]	p.[Thr108Met];[His121Asn]	p.[His121Asn];[Ala139Val]	p.[Arg130Trp];[Tyr208Cys]	p.[Lys202del];[Asp177Tyr]	p.[His121Asn];[His121Asn]	p.[Lys202del];[Ser51Ilefs*99]	p.[Arg130Trp]; [Lys202del]
mtDNA depletion (% depleted)	70%	75%	73%	85%	70-75%	55%. Multiple deletions	90%	60%. Multiple deletions
TK2 activity in pmol product/min /mg prot (% respect to controls]*	0.3 (2.9)	n.a.	n.a.	1.7 (17.5)	1.4 (14.3)	7.5 (68.5)	0.7 (6.3)	1.3 (14.1)

Table S1. Clinical, biochemical, and molecular features of a cohort of eight paediatric patients with TK2 deficiency. Patients were ordered depending on their age at the onset of the disease, from youngest (case 1) to oldest (case 8).
